# Supplementary material for: Increasing robustness of pairwise methods for effective connectivity in magnetic resonance imaging by using fractional moment series of BOLD signal distributions
Source: Netw Neurosci. 2019 Sep 1;3(4):1009–37. doi: 10.1162/netn_a_00099 (PMC6779268; doi:10.1162/netn_a_00099)
Supplement: Supplementary file 1 [file netn-03-1009-s001.pdf]

Bielczyk, N.Z., Llera, A., Buitelaar, J.K., Glennon, J.C., Beckmann, C.F. (2019). Supporting information for "Increasing robustness of pairwise methods for effective connectivity in Magnetic Resonance Imaging by using fractional moment series of BOLD signal distributions." *Network Neuroscience*, *3*(4), 1009–1037. https://doi.org/10.1162/netn_a_00099

Supplementary Materials

Supplementary material 1 DCM forward model

In this work, we use the original, single node per region DCM (Friston et al., 2003, Smith et al., 2011). This model operationalizes the generation of BOLD response from the neuronal networks across two levels: non-observable neuronal level and the observable hemodynamic level.

The latent neuronal dynamics is described by the simple differential relationship:

$$\frac{d\vec{z}}{dt} = A\vec{z}(t-\tau) + C\vec{u}(t) + \vec{\sigma}(t) \quad (6)$$

where z denotes the temporary activity across all nodes, u(t) denotes binary inputs (trains of on- and off- states in our case), A denotes the adjacency matrix of effective connectivity and C denotes connections from (experimental) inputs to the nodes, denotes a lag in the neuronal communication, and σ(t) denotes the level of stochasticity on the neuronal level. In our network setup, the modulatory connectivity does not play a role for the research question, therefore we set all the modulatory connections B from the original DCM model (Friston et al., 2003) to zero. The connectivity (a.k.a. adjacency) matrix A contains self-inhibition in every node as originally proposed in (Friston et al., 2003). Additionally, we use small, biologically plausible time lags of 50 [ms] in the communication between areas as also implemented in (Smith et al., 2011), therefore the simulated network becomes a system of delayed differential equations in fact.

In this context, the stochastic term σ(t) represents neuronal innovations which are not the part of the communication between nodes of the investigated network. It can either represent intrinsic dynamics in the given node (other than inhibition), or input from areas outside the investigated network. Strictly speaking, these innovations are not a 'noise' (which would mean stochasticity added to the neuronal time series on the top of the simulated dynamics), but rather a background neuronal

dynamics which cannot be explained by the given model. However, for the sake of simplicity we will refer to σ(t) as noise in the text below.

The observational level is given by the classic model for the hemodynamic response, referred to as Balloon-Windkessel model (Buxton et al., 1998, Friston et al., 2003), is described node-wide, and for every node *i* it is described by the dynamics of four biophysiological variables as follows:

$$\frac{ds_i}{dt} = z_i(t) - \kappa_i s_i(t) - \gamma_i(f_i(t) - 1)$$

$$\frac{df_i}{dt} = s_i(t) \qquad (7)$$

$$\lambda_i \frac{dv_i}{dt} = f_i(t) - v_i^{1/\alpha}(t)$$

$$\lambda_i \frac{dq_i(t)}{dt} = f_i(t)\frac{E(f_i(t), \rho_i)}{\rho_i} - v_i^{\frac{1}{\alpha}-1}(t)q_i(t)$$

where $s_i(t)$ - vasodilatory signal, $f_i(t)$ - inflow, $v_i(t)$ - blood volume, $q_i(t)$ - deoxyhemoglobin content, $E(f, \rho) = 1 - (1 - \rho)^{1/f}$. The model involves five node-specific constants: κ - rate of signal decay, γ - rate of flow-dependent elimination, λ - hemodynamic transit time, α - Grubb's exponent, ρ - resting oxygen extraction fraction. Then, the following expression describes the outcome BOLD response:

$$y(t) = V_0\left(7\rho_i\left(1 - q_i(t)\right)\right) + 2\left(1 - \frac{q_i(t)}{v_i(t)}\right) + (2\rho_i - 0.2)(1 - v_i(t)) \quad (8)$$

Inputs to the network were simulated as in [Smith et al., 2011]: as independent trains of on- and off-states with time resolution of TR = 5 [ms]. The probability of state switches was governed by a Poissonian process of a mean on-state duration of 2.5 [s], and a mean off- state duration of 7.5 [s].

Supplementary material 2 Benchmark synthetic datasets

Benchmark datasets by Smith et al., 2011 are build using the DCM forward model described in Supplementary Material 1. All the variations of the connectivity used in this study are acyclic and sparse, and only the size varies between 5 and 50 nodes.

The DCM forward model allows for emulating a variety of experimental conditions, such as the number of nodes in the network (N), the session duration (SD), the time resolution of the data (TR), the amount of thermal noise added to the BOLD response or the variability in delay of the hemodynamic response. In some simulations, additional features were introduced, e.g., shared inputs or backward connections. All the parameters are summarized in Table 1.

no	N	S D (min)	TR (s)	noise (%)	std of the HRF	Other features
1	5	10	3.0	1.0	0.5	
2	10	10	3.0	1.0	0.5	
3	15	10	3.0	1.0	0.5	
4	50	10	3.0	1.0	0.5	
5	5	60	3.0	1.0	0.5	
6	10	60	3.0	1.0	0.5	
7	5	250	3.0	1.0	0.5	
8	5	10	3.0	1.0	0.5	shared inputs
9	5	250	3.0	1.0	0.5	shared inputs
10	5	10	3.0	1.0	0.5	global mean confound
11	10	10	3.0	1.0	0.5	bad ROIs (time series mixedwith each other)

12	10	10	3.0	1.0	0.5	bad ROIs (new random time series mixed in)
13	5	10	3.0	1.0	0.5	backwards connections
14	5	10	3.0	1.0	0.5	cyclic connections
15	5	10	3.0	0.1	0.5	stronger connections
16	5	10	3.0	1.0	0.5	more connections
17	10	10	3.0	0.1	0.5	
18	5	10	3.0	1.0	0.0	
19	5	10	0.25	0.1	0.5	neural lag=100 ms
20	5	10	0.25	0.1	0.0	neural lag=100 ms
21	5	10	3.0	1.0	0.5	2-group test
22	5	10	3.0	0.1	0.5	nonstationary connection strengths
23	5	10	3.0	0.1	0.5	stationary connection strengths
24	5	10	3.0	0.1	0.5	only one strong external input
25	5	5	3.0	1.0	0.5	
26	5	2.5	3.0	1.0	0.5	
27	5	2.5	3.0	0.1	0.5	
28	5	5	3.0	0.1	0.5	

Table 1: All the parameters included in the benchmark synthetic datasets (Smith et al., 2011)

Supplementary material 3 Complex cumulants, a 2-node simulation in polar coordinates

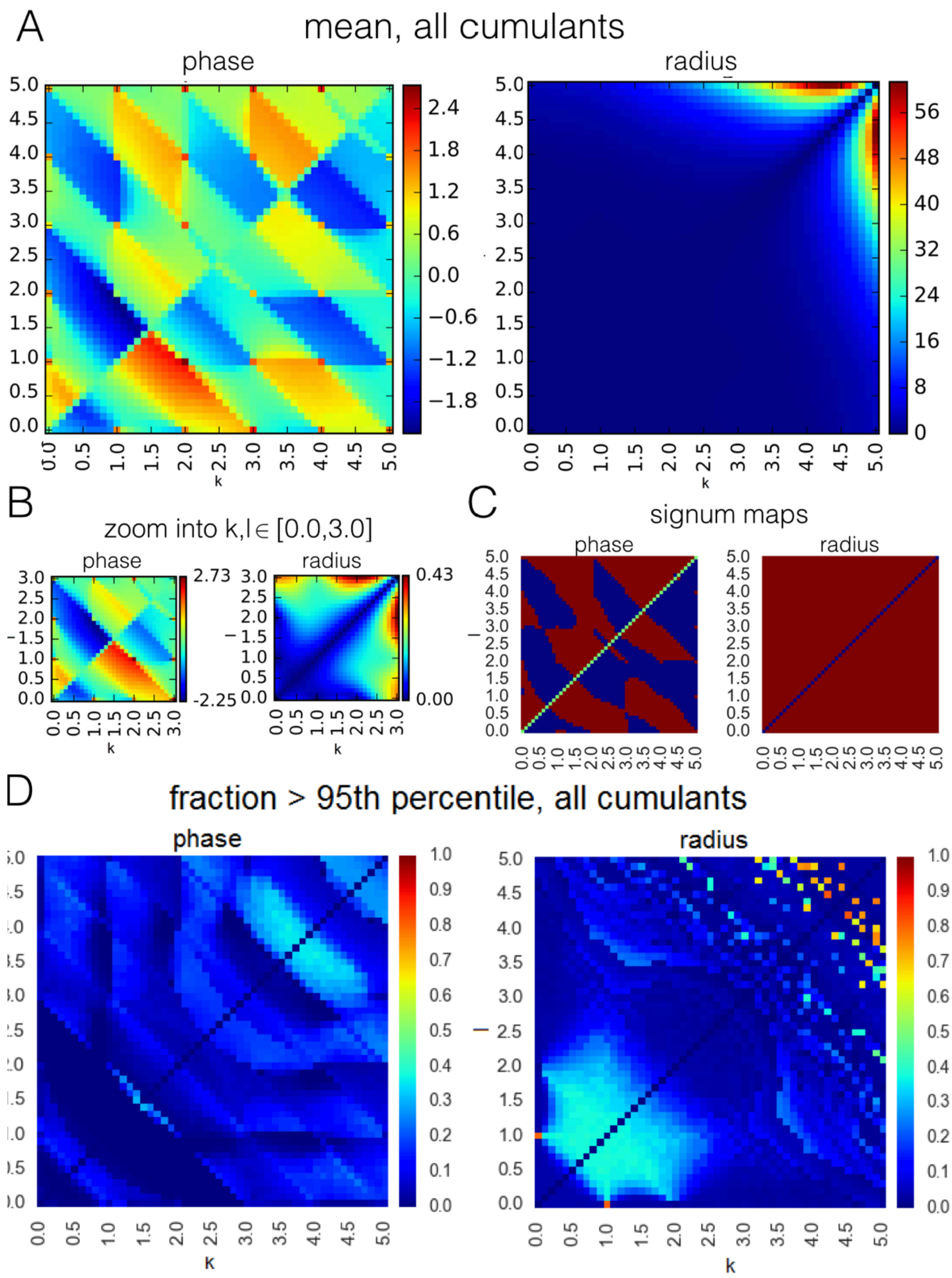

Figure 9 Cumulants in polar coordinates. A: mean values for all cumulants, over 1000 simulations. The radius and the phase are computed with respect to point (0, 0), otherwise the (anti-)symmetry would be lost. Since cumulants are antisymmetric with respect to indexing k, l, the heatmaps for the phase are antisymmetric. The radius is always positive. B: zoom into a smaller range of [0.0; 3.0]. C: signum of the cumulants. Red: positive. Blue: negative. Green: zero. D: Discriminative power for all cumulants in range k, l in [0:0; 5:0], in polar coordinates (in the ideal case of a very long BOLD time series and no background neuronal noise). Cumulants in polar coordinates are less informative. Phase is uninformative since the discriminative power is relatively high only in the high-moment regime - and high moments are

Supplementary material 4 Success rate for all the cumulants in the synthetic benchmark datasets

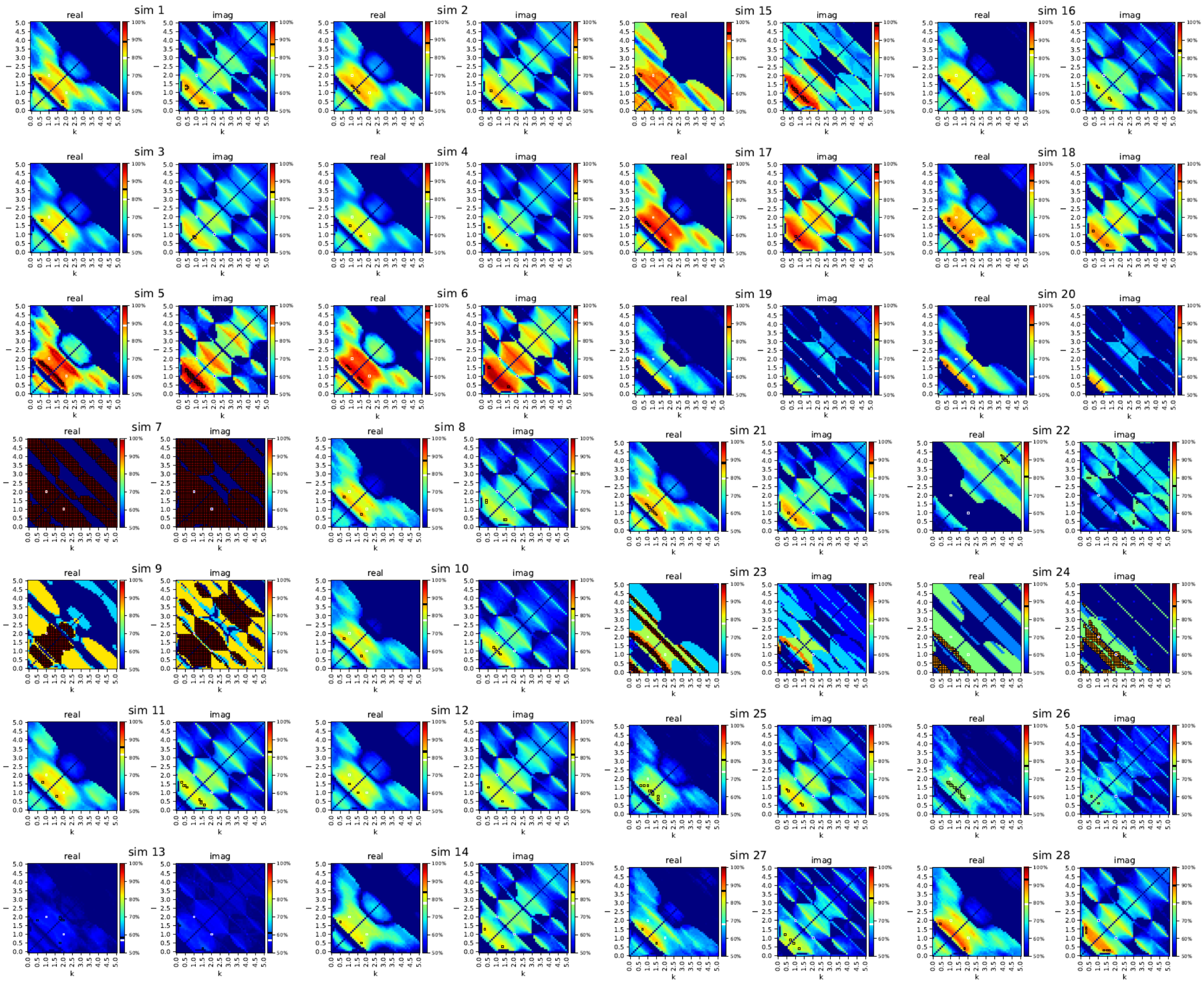

Figure 10 Success rate for all the individual cumulants, for the remaining 27 simulations at benchmark datasets. In every case, there are cumulants performing better than the cumulant (2,1). Simulation no 7 is particularly convenient for effective connectivity methods whereas simulation no 13 is particularly challenging.

In Fig. 10, results for the remaining 27 simulations from Smith el al., 2011 are presented. For all the methods, simulation no 7 is particularly convenient for effective connectivity methods as it contains the most convenient choice of parameters: the network is small (N=5) and the simulation is unrealistically long (T = 250 [min]). As discussed in Smith et al., 2011, simulation length is one of the crucial factors influencing methods for effective connectivity. Simulation 13 is particularly hard on

the other hand. In this simulation, each adjacency matrix additionally contains negative (inhibitory) backwards connections for a randomly chosen half of all connections (connectivity strength for new, backwards connections is sampled from the same distribution as the original connections). With inhibitory connections confounding the influence of exhibitory connections, none of the tested methods achieved mean performance higher than 60% success rate.

Supplementary material 5 Comparison between methods on all the synthetic benchmark datasets

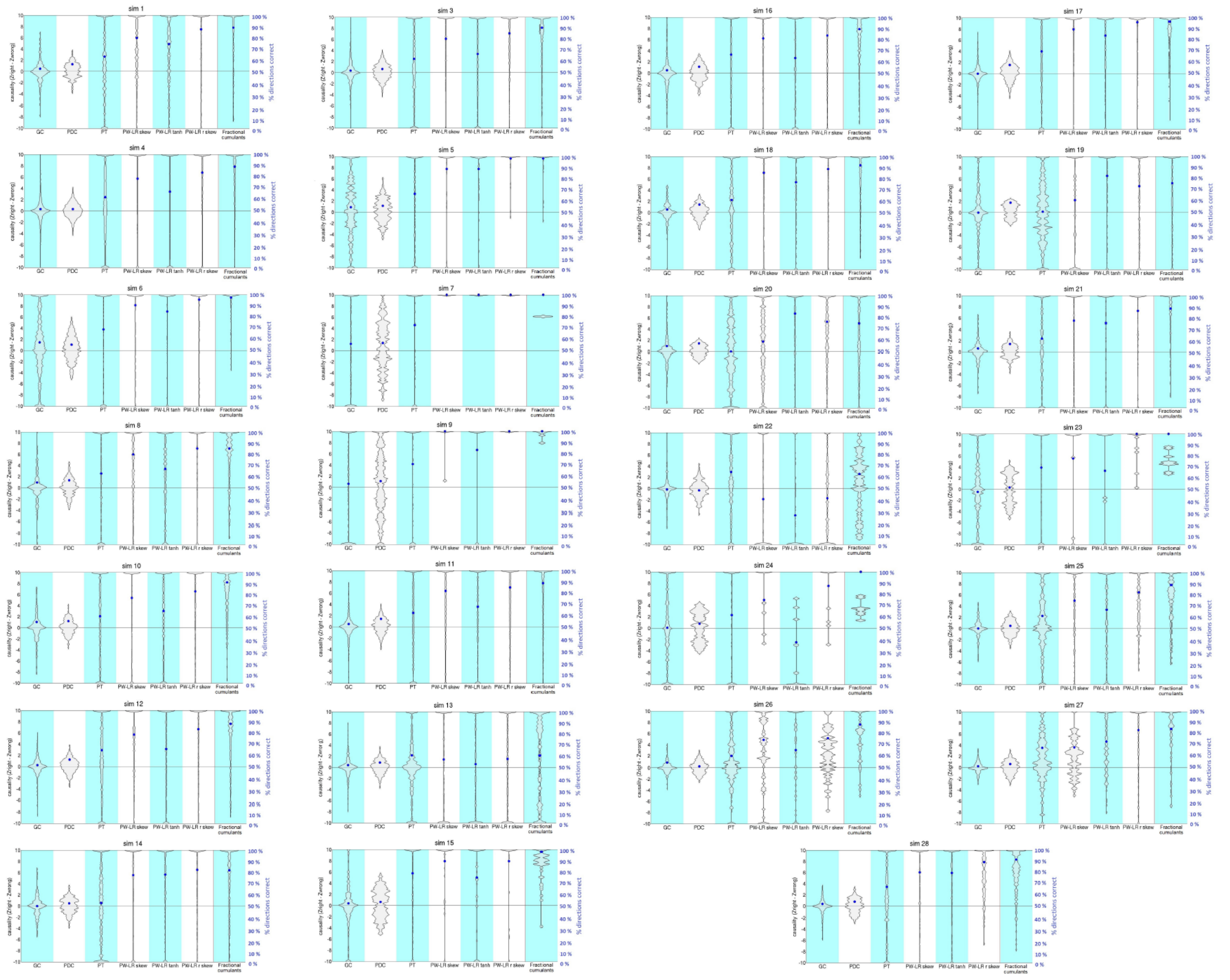

Figure 11 Overall success rate of Fractional cumulants against 'PW-LR' methods. In most of the simulations, we achieved a slight improvement with respect to the main competitor, 'PW-LR r skew'. In simulations 7, 8, 9, 14, 17, 20 and 23, the performance are roughly the same.

Supplementary material 6 'Net connectivity': Cumulant maps for a simple two-node system, in case of a uni- versus bidirectional connection

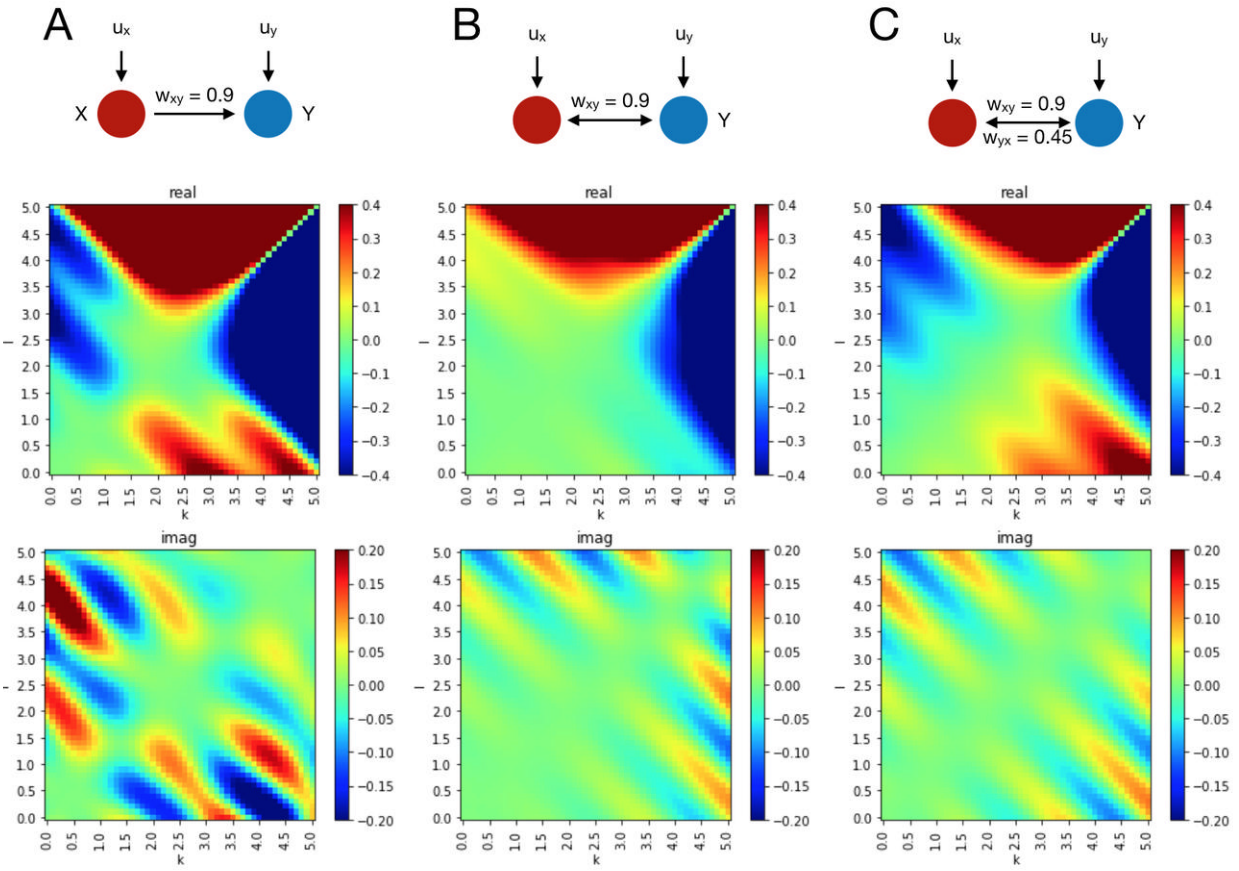

Figure 12 Net connectivity effect. Simulation of a 10 [min] dynamics of a two-node linear system, Eq. 5, Fs = 200 [Hz], σ = 0, binary inputs simulated as stochastic trains of on- and off-states as described in the Methods section. A: unidirectional connection with connection weight w = 0.9, B: bidirectional connection with weights w_{XY} = w_{YX} = 0.9, C: asymmetric bidirectional connection with weights w_{XY} = 0.9, w_{YX} = 0.45. When the connection is perfectly symmetrical, B, the amplitudes of cumulants will be *weaker* than in case of an asymmetric, unidirectional connection, A. In case of bidirectional connection with *asymmetric weights* though, C, the cumulant maps will give more pronounced effect than in case of a perfectly bidirectional connection, B and weaker than in case of perfectly unidirectional connection, A. As a conclusion, what the cumulant maps reflect is the *difference* between two connectivity strengths X → Y and Y → X.

In Fig. 12, results of a simple simulation of a two-node linear system (simulated with ordinary differential equations, Eq. 5, 10 [min] simulation with Fs = 200 [Hz], $\sigma = 0$, binary inputs simulated as stochastic trains of on- and off- states as described in the Methods section) are presented. We simulated the same system with the same set of inputs in three scenarios: (A) unidirectional connection with connection weight w = 0.9, (B) bidirectional connection with weights $w_{XY} = w_{YX} = 0.9$, (C) asymmetric bidirectional connection with weights $w_{XY} = 0.9$, $w_{YX} = 0.45$.

The results demonstrate that, in case the connection is perfectly symmetrical, B, the amplitudes of cumulants will be *weaker* than in case of an asymmetric, unidirectional connection, A. In case of bidirectional connection with *asymmetric weights* though, C, the cumulant maps will give more pronounced effect than in case of a perfectly bidirectional connection, B (as the system of less symmetrical in case), but at the same time, weaker than in case of perfectly unidirectional connection, A. As a conclusion, what the cumulant maps reflect is the *difference* between two connectivity strengths X → Y and Y → X. We refer to this effect as *net connectivity*.
